# Oral Administration of Surface-Deacetylated Chitin Nanofibers and Chitosan Inhibit 5-Fluorouracil-Induced Intestinal Mucositis in Mice

**DOI:** 10.3390/ijms18020279

**Published:** 2017-01-27

**Authors:** Ryo Koizumi, Kazuo Azuma, Hironori Izawa, Minoru Morimoto, Kosuke Ochi, Takeshi Tsuka, Tomohiro Imagawa, Tomohiro Osaki, Norihiko Ito, Yoshiharu Okamoto, Hiroyuki Saimoto, Shinsuke Ifuku

**Affiliations:** 1 Graduate School of Engineering, Tottori University, Tottori 680-8552, Japan; M16T4009Y@edu.tottori-u.ac.jp (R.K.); h-izawa@chem.tottori-u.ac.jp (H.I.); saimoto@chem.tottori-u.ac.jp (H.S.); 2 Department of Veterinary Clinical Medicine, Tottori University, Tottori 680-8553, Japan; kosukeochi28@gmail.com (K.O.); tsuka@muses.tottori-u.ac.jp (T.T.); imagawat@muses.tottori-u.ac.jp (T.I.); tosaki@muses.tottori-u.ac.jp (T.O.); taro@muses.tottori-u.ac.jp (N.I.); yokamoto@muses.tottori-u.ac.jp (Y.O.); 3 Division of Instrumental Analysis, Research Center for Bioscience and Technology, Tottori University, Tottori 680-8550, Japan; morimoto@chem.tottori-u.ac.jp

**Keywords:** surface deacetylated chitin nanofiber, chitosan, mucositis, 5-fluorouracil, apoptosis

## Abstract

This study investigated the prophylactic effects of orally administered surface-deacetylated chitin nanofibers (SDACNFs) and chitosan against 5-fluorouracil (5-FU)-induced intestinal mucositis, which is a common side effect of 5-FU chemotherapy. SDACNFs and chitosan abolished histological abnormalities associated with intestinal mucositis and suppressed hypoproliferation and apoptosis of intestinal crypt cells. These results indicate that SDACNF and chitosan are useful agents for preventing mucositis induced by anti-cancer drugs.

## 1. Introduction

Intestinal mucositis is a common side effect of anti-cancer drugs [1]. The antimetabolite anticancer agent 5-fluorouracil (5-FU) is widely used to treat malignancies as it improves tumor-free status and survival rates [2]. However, 50%–80% of patients treated with 5-FU exhibit mucositis as a side effect, with symptoms including severe diarrhea [3,4,5]. This often necessitates a reduction in drug dosage or even discontinuation of the treatment, which limits the success of cancer chemotherapy.

Chitosan is a linear polysaccharide of the chitin family derived from partial deacetylation of chitin [6]. It is a copolymer comprising (1 → 4)-2-acetamido-2-deoxy-β-d-glucan (*N*-acetyl-d-glucosamine) and (1 → 4)-2-amino-2-deoxy-β-d-glucan (d-glucosamine) units distributed throughout the biopolymer chain either randomly or as a block, depending on the specific method of deacetylation [7]. Chitosan has diverse pharmaceutical and biomedical applications due to its availability, non-toxicity, biocompatibility, biodegradability, and broad range of functionalities [7]. In fact, chitosan was shown to prevent gastrointestinal toxicity induced by 5-FU or doxorubicin without interfering with the anti-cancer effects [8,9].

Simple methods for preparing chitin nanofibers have recently been established [10,11,12]. In surface-deacetylated chitin nanofibers (SDACNFs), the nanofiber surface was transformed into chitosan by deacetylation while the core was maintained as a chitin crystal [13]. Owing to cationic electrostatic repulsive forces, the deacetylated chitin readily disintegrated and the SDACNFs were homogeneously dispersed in water. We have reported that SDACNFs have various biological activities, including effects on obesity and hypercholesterolemia by oral administration [14,15] and on wound healing by topical application [16,17]. In this study, we investigated the prophylactic effects of orally administered SDACNFs against 5-FU-induced intestinal mucositis.

## 2. Results and Discussion

### 2.1. SDACNFs Suppress the Effects of 5-FU-Induced Intestinal Mucositis

**Figure 1:**
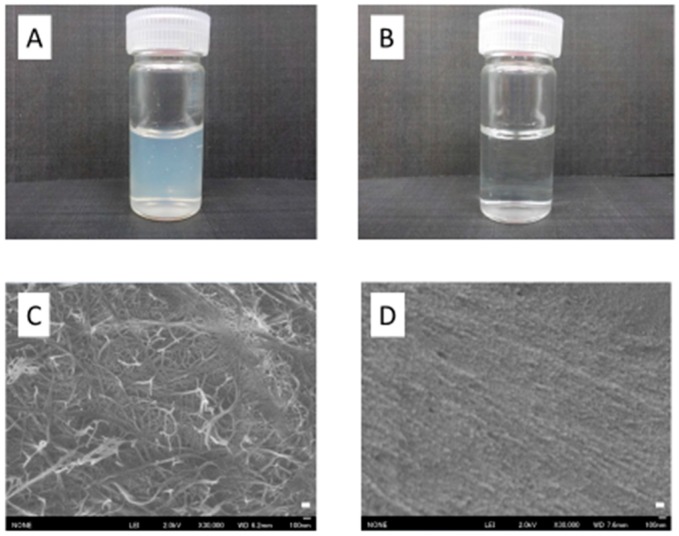
Low-magnification images and scanning electron micrographs of SDACNF and chitosan. (**A**,**B**) gross appearance of SDACNF (**A**) and chitosan (**B**); (**C**,**D**) scanning electron micrographs of SDACNF (**C**) and chitosan (**D**). Scale bar indicate 100 nm.

**Figure 2:**
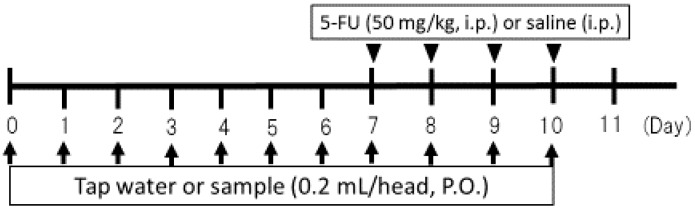
Schema of animal experiments. i.p.: intraperitoneal injection, P.O.: per os (oral administration).

**Figure 3:**
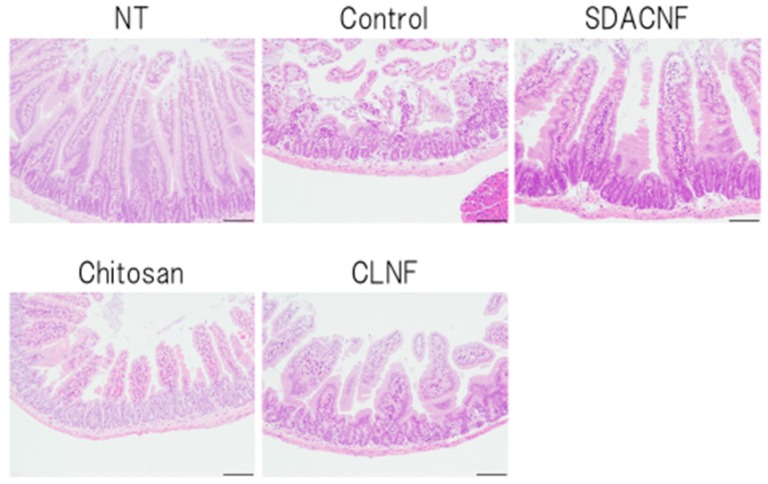
Histopathological analysis of intestinal crypt tissue. Images were obtained from one of three mice per group. Low magnification images are shown. Bar: 100 μm. NT: No treatment; Control: control group; SDACNF: surface deacetylated chitin nanofiber group; Chitosan: chitosan group; CLNF: cellulose nanofiber group.

Low-magnification images and scanning electron micrographs of SDACNF and chitosan are shown in Figure 1. Chitosan was dissolved in 0.5% acetic acid, whereas SDACNF was a gel that formed a nanofiber network. The study design is shown schematically in Figure 2. There were no differences in small intestine length and weight-to-length ratio among control, SDACNF, chitosan, and cellulose nanofiber (CLNF) groups (data not shown). In the latter two groups, mucosal architecture, ulceration of the mucosa, reduction in villus height, and inflammatory cell infiltration (MI) were observed by hematoxylin and eosin (H&E) staining (Figure 3). However, these were not detected in the chitosan and SDACNF groups.

**Figure 4:**
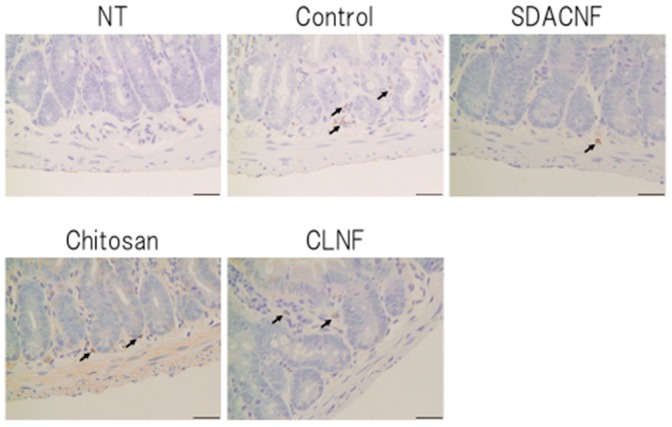
Immunohistochemical analysis of MPO activity. Images were obtained from one of three mice per group. Black arrows indicate MPO-positive cells. High magnification images are shown. Bar: 50 μm. NT: No treatment; Control: control group; SDACNF: surface deacetylated chitin nanofiber group; Chitosan: chitosan group; CLNF: cellulose nanofiber group.

**Figure 5:**
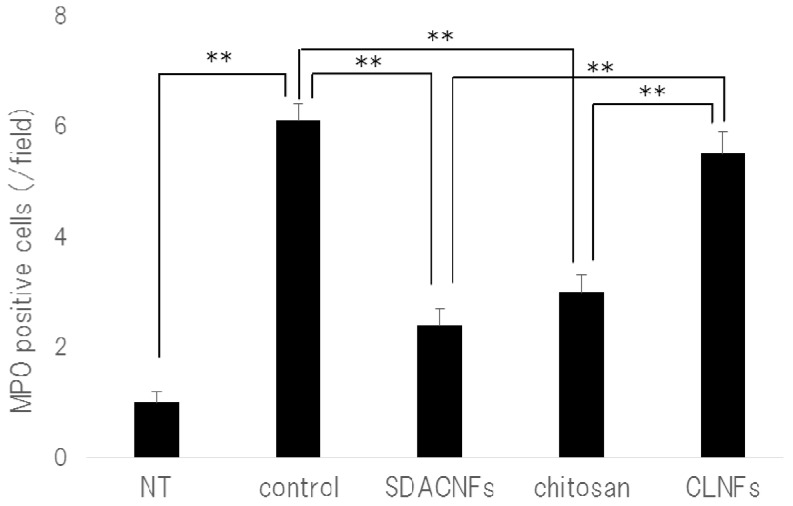
Number of MPO-positive cells. Data represent mean ± S.E. Means were compared with the Steel–Dwas test. ** *p* < 0.01. MPO-positive cells were counted under the microscope in 10 randomly selected high-power fields (200× magnification) per cross section. The jejunum of three mice per group were analyzed. The mean number of MPO-positive cells in 30 fields was determined for each group. NT: No treatment; Control: control group; SDACNF: surface deacetylated chitin nanofiber group; Chitosan: chitosan group; CLNF: cellulose nanofiber group.

Different aspects of intestinal architecture in each group were assigned a histological score based on H&E staining (Table 1). The overall score as well as scores for mucosal ulceration and inflammation and villus height were lower in the SDACNF than in the control and CLNF groups. In the chitosan group, the score for mucosal inflammation was lower than that of control mice, and there were fewer myeloperoxidase (MPO)-positive cells in the SDACNF (2.4 ± 0.3 cells/field) and chitosan (3.0 ± 0.3 cells/field) groups than in the control (6.1 ± 0.3 cells/field) and CLNF (5.5 ± 0.4 cells/field) groups (each *p* < 0.01, Figure 4 and Figure 5).

Neutrophils are the first cells that arrive at the site of inflammation, and they induce an increase in myeloperoxidase (MPO) activity in the first hours after 5-FU administration [18,19]. Intestinal mucositis is characterized histologically by a shortening of intestinal villi and disruption of intestinal crypts [3,4,5]. Control mice in the present study exhibited intestinal mucositis; in contrast, this was suppressed in the SDACNF and chitosan groups, indicating that oral administration of SDACNF and chitosan inhibits 5-FU-induced histological damage associated with mucositis.

### 2.2. SDACNF Prevents Apoptosis of Intestinal Crypt Cells Induced by 5-FU

**Figure 6:**
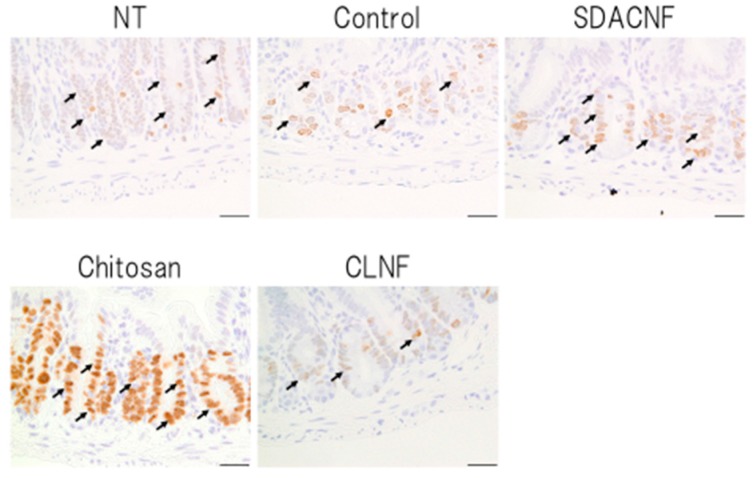
Immunohistochemical analysis of Ki-67 expression. Images were obtained from one of three mice per group. Black arrows indicate Ki-67-positive cells. High magnification images are shown. Bar: 50 μm. NT: No treatment; Control: control group; SDACNF: surface deacetylated chitin nanofiber group; Chitosan: chitosan group; CLNF: cellulose nanofiber group.

**Figure 7:**
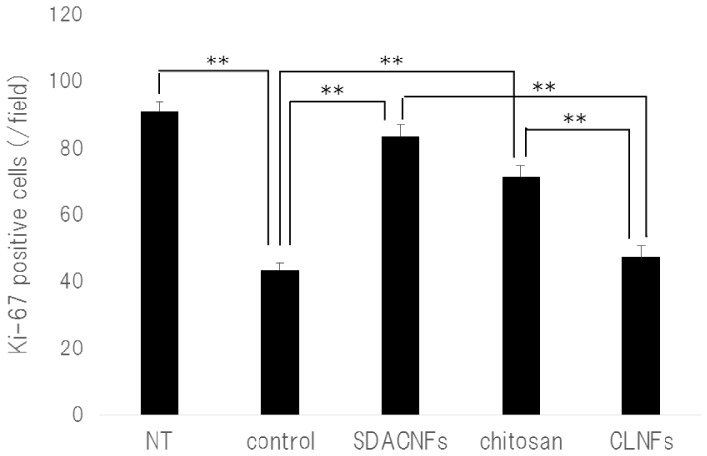
Number of Ki-67 positive cells. Data are expressed mean ± S.E. Means were compared with the Steel–Dwas test. ** *p* < 0.01. Ki-67 positive cells were counted under the microscope in 10 randomly selected high-power fields (200× magnification) per cross section. The jejunum of three mice per group were analyzed. The mean number of Ki-67-positive cells in 30 fields was determined for each group. NT: No treatment; Control: control group; SDACNF: surface deacetylated chitin nanofiber group; Chitosan: chitosan group; CLNF: cellulose nanofiber group.

**Figure 8:**
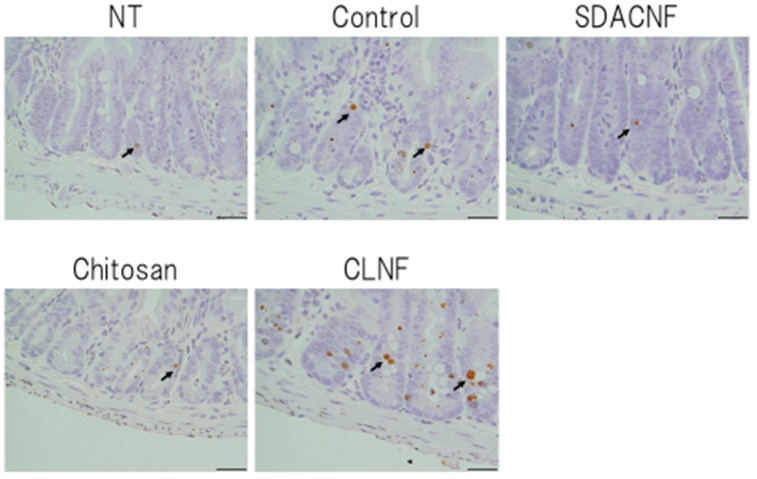
Detection of apoptotic cells with the TUNEL assay. Images were obtained from one of three mice per group. Arrows indicate TUNEL-positive cells. High magnification images are shown. Bar: 50 μm. NT: No treatment; Control: control group; SDACNF: surface deacetylated chitin nanofiber group; Chitosan: chitosan group; CLNF: cellulose nanofiber group.

**Figure 9:**
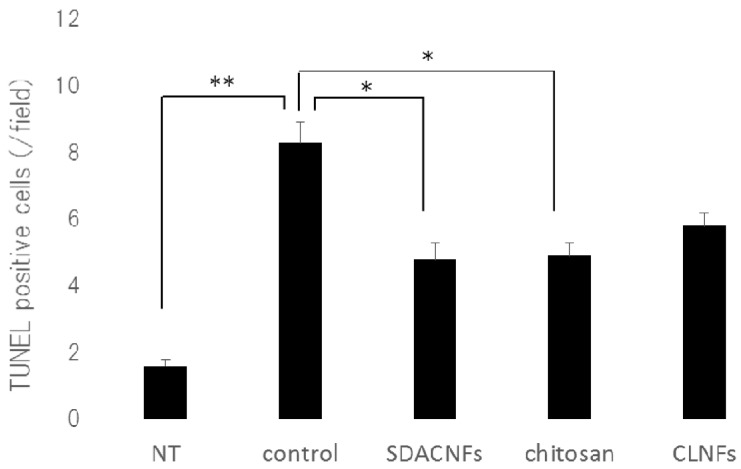
Number of TUNEL positive cells. Data represent mean ± S.E. Means were compared with the Steel–Dwas test. * *p* < 0.05, ** *p* < 0.01. TUNEL-positive cells were counted under the microscope in 10 randomly selected high-power fields (200× magnification) per cross section. The jejunum of three mice per group were analyzed. The mean number of TUNEL-positive cells in 30 fields was determined for each group. NT: No treatment; Control: control group; SDACNF: surface deacetylated chitin nanofiber group; Chitosan: chitosan group; CLNF: cellulose nanofiber group.

**Figure 10:**
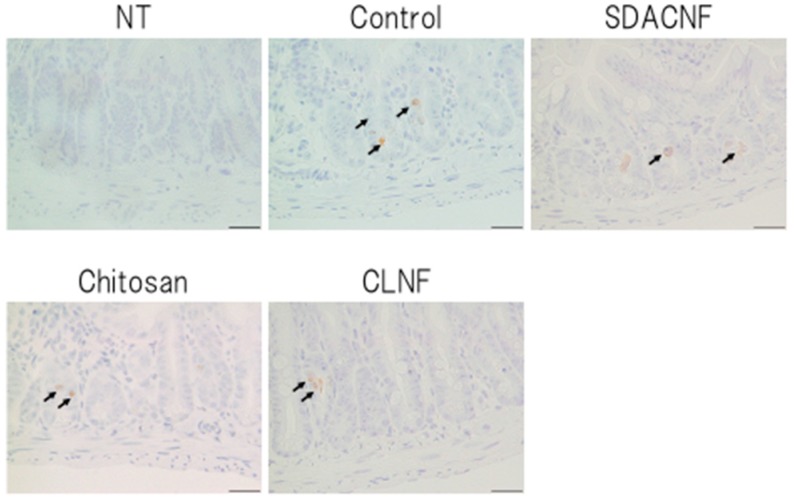
Immunohistochemical analysis of caspase-3 expression. Images were obtained from one of three mice per group. Arrows indicate caspase-3-positive cells. High magnification images are shown. Bar: 50 μm. NT: No treatment; Control: control group; SDACNF: surface deacetylated chitin nanofiber group; Chitosan: chitosan group; CLNF: cellulose nanofiber group.

**Figure 11:**
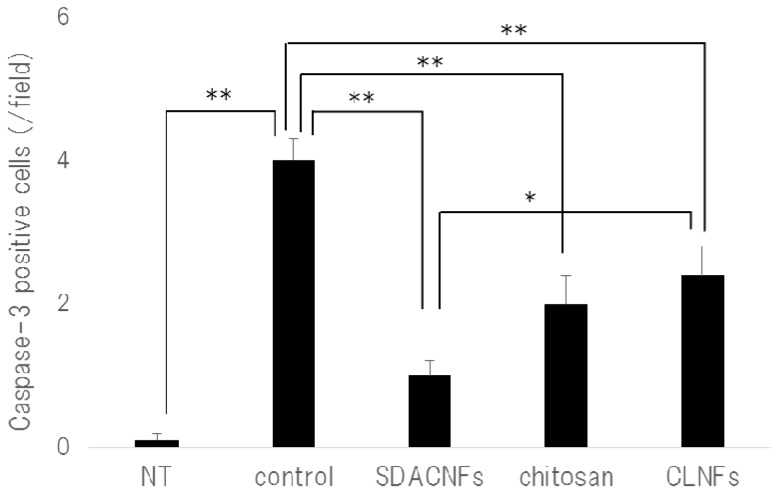
Number of caspase-3 positive cells. Data represent mean ± S.E. Means were compared with the Steel–Dwas test. * *p* < 0.05, ** *p* < 0.01. Caspase-3-positive cells were counted under the microscope in 10 randomly selected high-power fields (200× magnification) per cross section. The jejunum of three mice per group were analyzed. The mean number of caspase-3 positive cells in 30 fields was determined for each group. NT: No treatment; Control: control group; SDACNF: surface deacetylated chitin nanofiber group; Chitosan: chitosan group; CLNF: cellulose nanofiber group.

The results of Ki-67 immunohistochemistry revealed greater numbers of Ki-67-positive cells in the crypt in the SDACNF (83.6 ± 3.4 cells/field) and chitosan (71.5 ± 3.3 cells/field) groups than in the control (43.5 ± 2.1 cells/field) and CLNF (47.3 ± 3.7 cells/field) groups (*p* < 0.01 each) (Figure 6 and Figure 7). On the other hand, there were fewer caspase-3-positive cells in the SDACNF (1.0 ± 0.2 cells/field), chitosan (2.0 ± 0.4 cells/field), and CLNF (2.4 ± 0.4 cells/field) groups than in the control (4.0 ± 0.3 cells/field) group (SDACNF vs. control and chitosan vs. control, *p* < 0.01; and CLNF vs. control, *p* < 0.05) (Figure 8 and Figure 9). This was consistent with the results of the terminal deoxynucleotidyl transferase dUTP nick-end labeling (TUNEL) assay, which showed fewer TUNEL-positive cells in the SDACNF (4.8 ± 0.5 cells/field) and chitosan (4.9 ± 0.4 cells/field) groups than in control (8.3 ± 0.6 cells/field) mice (*p* < 0.05 each) (Figure 10 and Figure 11).

Although the pathogenic mechanism of intestinal mucositis induced by anti-cancer drugs is not well understood, it is thought to be a consequence of various processes such as apoptosis and abnormal inflammation in the intestinal mucosa as well hypoproliferation of intestinal crypt cells [20,21,22]. Ki-67 is a marker of proliferating cells that is expressed during all active phases of the cell cycle [23]. Our results indicate that oral administration of SDACNF and chitosan suppresses the crypt cell hypoproliferation induced by 5-FU.

Apoptosis is an important consequence of intestinal mucositis induced by anti-cancer drugs such as 5-FU [24,25,26,27]; indeed, we observed an increase in the number of TUNEL- and caspase-3-positive apoptotic cells in the intestinal crypt of control mice, which was abolished in the SDACNF and chitosan groups. Caspase-3 is the main downstream effector in apoptosis [28,29], whose activity was shown to be increased during intestinal cell apoptosis induced by chemotherapy, including 5-FU treatment [30,31,32]. Our results indicate that oral administration of SDACNF and chitosan suppressed apoptosis in the intestinal crypt by inhibiting caspase-3 activity. Although the suppression of apoptosis by chitosan has been previously demonstrated [33], ours is the first report describing the anti-apoptotic effects of orally administered chitosan in intestinal mucositis. The effectiveness of chitosan-derived gel has been previously described [34]. However, its mechanism of action is unknown. We have demonstrated that SDACNFs have similar bioactivity [14,15], and the results of the present study indicate that SDACNFs are equally or more effective than chitosan in preventing intestinal mucositis. However, the mode of absorption and metabolites of SDACNFs remain unknown and require clarification in future studies.

## 3. Materials and Methods

### 3.1. Animals and Regents

Female C57BL/6 mice (5 weeks old) were purchased from CLEA Japan (Osaka, Japan) and maintained under standard conditions in a room with constant temperature (22 ± 2 °C) and humidity (50% ± 5%) on a 12:12-h light/dark cycle (lights on, 7:00–19:00 h). Mice had free access to food (CE-2; CLEA Japan) and tap water during the experimental period. The animal procedures in this study were approved by the Animal Research Committee of Tottori University (15-T-2, from 1 April 2015 to 31 March 2016).

Chitin (Chitin TC-L) and chitosan (Chitosan FH-80) powder from crab shell were purchased from Koyo Chemical Co. (Tokyo, Japan). CLNF was purchased from Sugino Machine Co. (BiNFi-s cellulose, WMa-10002; Sugino Macine Limited, Uodu, Japan). Samples were used at concentrations of 1.0 *wt* % in 0.5 *wt* % aqueous acetic acid.

### 3.2. Preparation of SDACNFs

SDACNFs were prepared as previously described [13], with some modifications. Chitin powder (45.0 g) was treated with 20% (*w*/*w*) NaOH (1.5 L) for 6 h under reflux and an argon atmosphere. After deacetylation, the supernatant was removed by decantation, and the precipitate was thoroughly washed with distilled water and 0.5 *wt* % aqueous acetic acid by centrifugation to remove water-soluble products of NaOH, sodium acetate, and alkaline hydrolyzed chitin. For mechanical disintegration, deacetylated chitin was dispersed in 4.0 L of aqueous acetic acid. The sample was passed twice through a grinder (MKCA6-3; Masuko Sangyo Co., Kawaguchi, Japan) at 1500 rpm. The concentration, yield, and degree of deacetylation of surface-deacetylated chitin NFs were 1.3%, 74%, and 20 *wt* %, respectively.

### 3.3. Study Design

Mice were randomized into five groups: untreated controls administered tap water without 5-FU injection; controls administered tap water with 5-FU injection; the SDACNF group administered SDACNF with 5-FU injection; the chitosan group administered chitosan with 5-FU injection; and the CLNF group administered CLNF with 5-FU injection. Intragastric administration of tap water or samples (0.2 mL/head) was carried out from day 0 to day 10 via a catheter. Intestinal mucositis was induced via single daily intraperitoneal injection of 50 mg/kg 5-FU (Kyowa Hakko Kirin Co., Tokyo, Japan) over the course of 4 days (days 7–10) after 2 h of oral administration of tap water or sample. Animals were sacrificed under deep anesthesia 24 h after final 5-FU injection (day 11); the small intestine (duodenum, jejunum, and ileum) was removed and the length and weight were measured. The jejunum was immersed overnight in 10% neutralized formalin for histological study.

### 3.4. Histological Analysis

Thin sections (4 μm) were cut from each sample for H&E staining and histological analysis. Each section was examined by microscopy, and histological scoring was carried out as previously described [19] based on alterations in the mucosal architecture (general structure, cell distribution, mucosal and submucosal aspects), ulceration, villus height, and inflammatory cell infiltration. The score ranged from 0 (no alteration) to 3 (severe alteration). Results are presented as the sum of scores obtained for each parameter. Scoring was based on 10 randomly selected fields at 100× magnification in three mice per group; mean scores for 30 fields yielded the histological score for each group.

### 3.5. Ki-67 Immunohistochemistry

Ki-67 immunohistochemistry was carried out according to our previous methods [35]. Tissue sections (4 μm) were placed on glass slides and deparaffinized, washed with ethanol and water, and immersed in phosphate-buffered saline (PBS). The sections were autoclaved in 0.01 M citrate buffer (pH 6.0) for 15 min at 121 °C, then washed with PBS and incubated for 30 min at room temperature with rabbit polyclonal anti-Ki-67 antibody (1:50, E0468; Dako, Glostrup, Denmark). After washes with PBS, the sections were incubated for 30 min at room temperature with rat anti-IgG (1:100, sc-372; Vector Laboratories, Burlingame, CA, USA), washed with PBS, stained for 30 min using the Vectastain ABC kit (PK-4000; Vector Laboratories), then counterstained with HistoGreen (Nichirei Bioscience, Osaka, Japan) followed by nuclear Fast Red. The number of Ki-67-positive cells in crypts was counted under the microscope in 10 randomly selected high-power fields (200× magnification) per cross section. The jejunum of three mice per group were analyzed. The mean number of Ki-67-positive cells in 30 fields was determined for each group.

### 3.6. Caspase-3 Immunohistochemistry

Tissue sections (4 μm) were placed on glass slides and deparaffinized, washed with ethanol and water, and immersed in PBS. The sections were autoclaved in 0.01 M citrate buffer (pH 6.0) for 10 min at 121 °C, washed with PBS and incubated with 0.3% H_2_O_2_ to quench intrinsic peroxidase activity. This was followed by incubation with anti-cleaved caspase-3 (Asp175) antibody (1:400, 9961S; Cell Signaling Technology, Danvers, MA, USA) for 60 min at room temperature. After washes with PBS, sections were incubated with Envision (anti-rabbit, K4003; Dako) for 30 min at room temperature. The slides were washed with PBS, and immunoreactivity was visualized with a diaminobenzidine kit (S2022; Dako). The number of caspase-3-positive cells in the crypt was counted as described above for Ki-67 immunohistochemistry.

### 3.7. TUNEL Assay

The TUNEL assay was carried out as previously described [36]. Tissue sections (3 μm) on glass slides were deparaffinized, washed with ethanol and water, and immersed in distilled water. TUNEL staining was performed using the In Situ Apoptosis Detection kit (Takara Bio, Otsu, Japan) according to the manufacturer’s instructions. The number of TUNEL-positive cells in the crypts was counted as described above for Ki-67 immunohistochemistry.

### 3.8. MPO Staining

MPO staining was performed according to standard procedures [37]. The number of MPO-positive cells in the crypt was counted as described above for Ki-67 immunohistochemistry.

### 3.9. Statistical Analysis

Data are expressed as the mean ± standard deviation. Statistical analyses were performed using the Steel–Dwas test. A *p*-value < 0.05 was considered statistically significant.

## 4. Conclusions

In conclusion, oral administration of SDACNF and chitosan suppressed mucosal inflammation and shortening of intestinal crypts as well as crypt cell apoptosis and hypoproliferation caused by 5-FU treatment. These results indicate that SDACNF and chitosan are useful agents for preventing mucositis induced by anti-cancer drugs.

## Figures and Tables

**Table 1:** Histological score. Data represent mean ± S.E. Each H&E section was scored according to mucosal architecture (MA; general structure, cell distribution, mucosal and submucosal aspects), ulceration (MU), inflammation (MI), and villus height (VI). The score ranged from 0 (no alteration) to 3 (severe alteration). * *p* < 0.05 vs. control group; ^†^ *p* < 0.05. NT: No treatment; Control: control group; SDACNF: surface deacetylated chitin nanofiber group; Chitosan: chitosan group; CLNF: cellulose nanofiber group.

	NT	Control	SDACNF	Chitosan	CLNF
MA	0.0 ± 0.0	1.8 ± 0.6	0.8 ± 0.6 *^,^^†^	1.1 ± 0.6	1.8 ± 0.8
MU	0.0 ± 0.0	1.8 ± 0.6	0.8 ± 0.6 *^,^^†^	1.2 ± 0.6	1.8 ± 0.6
MI	0.0 ± 0.0	1.9 ± 0.5	0.7 ± 0.5 *^,^^†^	0.9 ± 0.5 *	1.5 ± 0.5
VI	0.0 ± 0.0	1.6 ± 0.7	0.8 ± 0.5 *^,^^†^	1.1 ± 0.7	1.8 ± 0.7
